# Counteraction of the multifunctional restriction factor tetherin

**DOI:** 10.3389/fmicb.2014.00163

**Published:** 2014-04-10

**Authors:** Daniel Sauter

**Affiliations:** Institute of Molecular Virology, Ulm University Medical CenterUlm, Germany

**Keywords:** ISG, restriction factor, tetherin, BST-2, HIV, Vpu, Nef, moonlighting proteins

## Abstract

The interferon-inducible restriction factor tetherin (also known as CD317, BST-2 or HM1.24) has emerged as a key component of the antiviral immune response. Initially, tetherin was shown to restrict replication of various enveloped viruses by inhibiting the release of budding virions from infected cells. More recently, it has become clear that tetherin also acts as a pattern recognition receptor inducing NF-κB-dependent proinflammatory gene expression in virus infected cells. Whereas the ability to restrict virion release is highly conserved among mammalian tetherin orthologs and thus probably an ancient function of this protein, innate sensing seems to be an evolutionarily recent activity. The potent and broad antiviral activity of tetherin is reflected by the fact that many viruses evolved means to counteract this restriction factor. A continuous arms race with viruses has apparently driven the evolution of different isoforms of tetherin with different functional properties. Interestingly, tetherin has also been implicated in cellular processes that are unrelated to immunity, such as the organization of the apical actin network and membrane microdomains or stabilization of the Golgi apparatus. In this review, I summarize our current knowledge of the different functions of tetherin and describe the molecular strategies that viruses have evolved to antagonize or evade this multifunctional host restriction factor.

## INTRODUCTION

In the late 1960s, researchers estimated that the human genome may contain up to two million protein-coding genes (Kauffman, 1969). Today, we know that the actual number is much lower. In 2012, an *in vitro* gene expression analysis yielded a number of 20,687 protein-coding genes (Pennisi, 2012). Interestingly, several hundred of them are interferon-stimulated genes (ISGs) that are upregulated during viral infections (de Veer et al., 2001; Lanford et al., 2006; Fernandez-Suarez et al., 2013). Although an antiviral effector function has been described for some of these factors, the role of most ISGs during viral infections remains obscure. Three extensively studied proteins induced by type I interferons (IFN) are the restriction factors APOBEC3G (apolipoprotein B mRNA-editing enzyme, catalytic polypeptide-like 3G), TRIM5α (tripartite motif 5-alpha), and tetherin (BST-2, CD317, or HM1.24). These host proteins are key players of the innate immune response and part of the first line of defense against lentiviruses. Like other IFN-inducible proteins, they target specific steps of the viral life cycle: APOBEC3G is a cytidine deaminase that inhibits reverse transcription and introduces G-to-A substitutions in the viral genome (Sheehy et al., 2002), TRIM5α binds incoming viral capsids and interferes with the uncoating process (Stremlau et al., 2004), and tetherin inhibits the release of budding virions from infected cells (Neil et al., 2008; Van Damme et al., 2008).

Surprisingly, the number of human genes is not significantly larger than that of many less complex organisms such as *Caenorhabditis elegans* or *Drosophila melanogaster*. Yet, the number of proteins can of course not be directly inferred from the number of genes. Alternative pre-mRNA splicing, DNA rearrangement, post-translational modifications, RNA editing, and the use of alternative start codons or reading frames are means to increase the coding potential of genes. Another possibility of coping with a limited number of genes is the evolution of multifunctional proteins, a phenomenon called “gene sharing” or “moonlighting” (Jeffery, 2003). ISGs may be especially prone to moonlighting since viruses exert a substantial selection pressure on the genomes of their host species. Antiviral proteins frequently have to acquire novel functions to cope with rapidly evolving or newly emerging viruses. Thus, viral infections may drive the evolution of antiviral activities in proteins that initially only exerted functions unrelated to immunity. Another common feature of many host restriction factors is their counteraction by viral antagonists. Whereas HIV-1 is resistant against human TRIM5α due to mutations in its capsid protein (Stremlau et al., 2004), APOBEC3G and tetherin are directly targeted by the accessory proteins Vif and Vpu, respectively (Sheehy et al., 2002; Neil et al., 2008; Van Damme et al., 2008). Notably, the combination of an antiviral function with activities beyond immunity within one protein may be a means to impede counteraction by viruses. Antiviral moonlighting proteins that also exert essential cellular functions cannot simply be degraded as this might be detrimental for the host cell and thus terminate viral replication.

The host restriction factor tetherin is such a moonlighting protein fulfilling all characteristics of known restriction factors: it is induced by IFNs, inhibits a specific step of the viral replication cycle, shows signatures of positive selection and is counteracted by viral proteins. In this review, I will summarize our current knowledge of the different activities of tetherin and discuss the strategies evolved by different viruses to antagonize or evade this restriction factor.

## STRUCTURE, TOPOLOGY, AND POST-TRANSLATIONAL MODIFICATIONS

**FIGURE 1:**
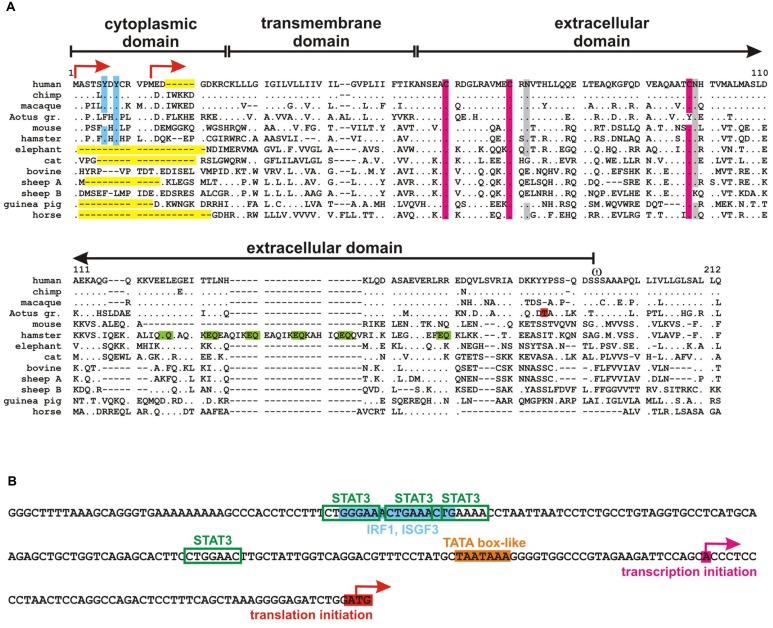
**Tetherin sequences. (A)** Alignment of tetherin orthologs from different mammalian species. Dots indicate identity and gaps (dashes) were introduced to improve the alignment. *Aotus gr.* designates the Gray-handed night monkey *Aotus griseimembra*, sheep A and B the two tetherin proteins encoded by sheeps. A dual tyrosine motif that has been shown to be important for NF-κB activation as well as RICH2 and adapter protein binding is highlighted in blue, conserved cystein residues that form disulfide bridges are shown in pink and glycosylation sites in gray. Threonine at position 164 of *Aotus* tetherin is responsible for the lack of virion retention in this species and is highlighted in red. A unique stretch of EQ repeats in the hamster ortholog that may determine Golgi localization is marked in green. Deletions and truncations in the N-terminal cytoplasmic domain including the human-specific five amino acid deletion are shown in yellow. Two alternative start codons are indicated by arrows and ω marks the omega site of GPI-anchor addition. **(B)** Partial sequence of the human tetherin promoter. Transcription and translation start sites are indicated by pink and red arrows, respectively. A TATA box-like sequence is highlighted in orange and STAT3 and IRF1/ISGF3 binding sites are marked in green and blue, respectively.

**FIGURE 2:**
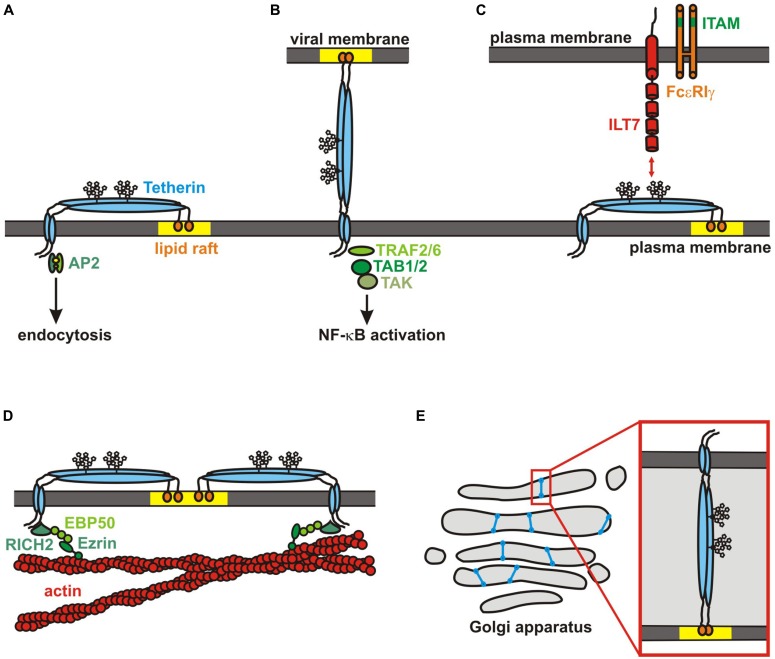
**Tetherin functions. (A)** Topology of a tetherin dimer: the N-terminal cytoplasmic domain is followed by an alpha-helical transmembrane domain, a glycosylated coiled-coil extracellular domain and the GPI-anchor that is located in lipid rafts (yellow). Adapter proteins bind to a dual tyrosine motif in the N-terminal domain to induce clathrin-mediated endocytosis. **(B)** A tetherin dimer restricting the release of a budding virion is shown. Whereas the GPI-anchor is incorporated into the viral membrane, the N-terminal transmembrane domain remains attached to the host cell. TRAFs are recruited to the N-terminus upon virion binding. Subsequent recruitment of TABs and activation of TAK1 induces an NF-κB-dependent antiviral immune response. **(C)** Tetherin interacts with ILT7 on pDCs. ILT7 forms a complex with FcεRIγ , which contains a cytoplasmic ITAM. **(D)** Tetherin forms a picket fence around lipid rafts and links them to the underlying actin cytoskeleton via RICH2/EBP50/Ezrin. **(E)** Hamster tetherin is required for maintenance of the Golgi apparatus. It may connect and stabilize opposite membranes in the Golgi cisternae or sense their curvature and distance.

The structural topology of tetherin is almost unique among mammalian proteins. Tetherin is a type II transmembrane protein consisting of a short N-terminal domain followed by an alpha-helical transmembrane domain, a labile coiled-coil ectodomain and a C-terminal glycosyl-phosphatidylinositol (GPI) anchor (**Figures 1A** and **2A**; Kupzig et al., 2003). This unusual topology with two membrane anchors is only shared with a special form of the prion protein (Hegde et al., 1998, 1999; Stewart et al., 2001), ponticulin from the slime mold *Dictyostelium discoideum* (Hitt et al., 1994a,b), Sm23 from *Schistosoma mansoni* (Köster and Strand, 1994), and NcSRS2 from *Neospora caninum* (Nishikawa et al., 2002).

The N-terminal intracellular domain of tetherin contains an evolutionarily conserved tyrosine motif (YxY; **Figure 1A**), which mediates clathrin-dependent internalization by recruitment of AP2 (**Figure 2A**; Ohtomo et al., 1999; Blasius et al., 2006; Rollason et al., 2007; Masuyama et al., 2009). Mature tetherin recycles between the plasma membrane, endosomes and the trans-Golgi-network (TGN) with a mean surface half-life of a few hours (Masuyama et al., 2009; Skasko et al., 2011; Sauter et al., 2013). Notably, methionine at position 13 of human tetherin has been shown to serve as alternative start codon, resulting in the expression of a shorter isoform (**Figure 1A**; Blasius et al., 2006; Cocka and Bates, 2012). Although both isoforms are expressed at comparable levels, the short isoform may preferentially localize to the plasma membrane due the lack of the YxY endocytosis motif (Cocka and Bates, 2012).

Mature tetherin is characterized by complex N-linked glycosylation at two conserved asparagine residues in its extracellular domain (**Figure 1A**; Ohtomo et al., 1999; Kupzig et al., 2003). Glycosylation occurs in the endoplasmic reticulum (ER) and Golgi apparatus and is required for entry of tetherin into the secretory pathway (Kupzig et al., 2003). Nevertheless, tetherin has never been shown to be secreted. Interestingly, glycosylated tetherin binds to various lectins with different selectivities (Ohtomo et al., 1999). However, a functional role of lectin binding by tetherin *in vivo* has not been demonstrated yet. In addition to the conserved asparagine residues, the ectodomain of human tetherin contains three cysteine residues that mediate disulfide-linked homodimer formation of the mature protein (**Figure 1A**; Kupzig et al., 2003). Recently, it has been suggested that interaction of the transmembrane domains may also be involved in homodimerization of tetherin (Cole et al., 2012). Tetherin homodimers are characterized by a parallel dimeric coiled-coil over the C-terminal two-thirds of the ectodomain. The N-terminal third may form an antiparallel four-helix bundle with another dimer, creating a tetherin tetramer (Schubert et al., 2010; Yang et al., 2010a).

During maturation, the C-terminus of tetherin is cleaved off in the ER to enable the addition of a GPI-anchor to serine at position 161 (**Figure 1A**). Tetherin is trapped in the ER in cells that have defects in the GPI-biosynthetic pathway (Perez-Caballero et al., 2009). The GPI-anchor serves as subcellular localization signal and targets tetherin to cholesterol-rich microdomains (lipid rafts; Kupzig et al., 2003). In contrast, the N-terminal transmembrane domain is most likely located outside lipid rafts with the cytosolic domain being a membrane microdomain exclusion motif (Billcliff et al., 2013). Thus, it has been suggested that parallel tetherin dimers form a picket fence like structure at the boundary of lipid rafts (**Figure 2D**; Kupzig et al., 2003).

## EXPRESSION AND INDUCTION

The tetherin core promoter has a size of about 2000 base pairs. Transcription starts 51 nucleotides upstream of the start codon and a TATA box-like sequence is located 81 nucleotides upstream of the start codon (**Figure 1B**; Ohtomo et al., 1999). The promoter contains consensus binding sites for the transcription factors STAT3, IRF1, and ISGF3 (**Figure 1B**), suggesting that tetherin is an IFN-inducible gene (Ohtomo et al., 1999; Kawai et al., 2006; Ge et al., 2006). Indeed, it has been shown that IFN-α, -β , -γ , -τ , -λ 3, and -ω induce the expression of tetherin in various cell types from different species (Blasius et al., 2006; Arnaud et al., 2010; Dietrich et al., 2011; Cobos Jiménez et al., 2012; Liu et al., 2012; Amet et al., 2014). Like other ISGs, tetherin expression is upregulated upon viral infection and protein levels correlate with viral loads both, in HIV-infected humans and simian immunodeficiency viruses (SIV)-infected macaques (Homann et al., 2011; Mous et al., 2012; Rahmberg et al., 2013). Retroviral infection is sensed by various pattern recognition receptors (PRR) such as TLRs, RIG-I, or IFI16 thereby inducing the release of type I IFNs and subsequent upregulation of tetherin (Blasius et al., 2006; Zhou et al., 2010; Jakobsen et al., 2013; Tavano et al., 2013; Wang et al., 2013). Interestingly, several IFN-independent stimulants of tetherin have also been reported. Bego et al. (2012) for example showed that TLR3 and TLR8 stimulation are able to induce the expression of tetherin independently of IFNs. Similarly, IL-27 has been identified as a potent inducer of tetherin in the absence of IFN (Guzzo et al., 2012). In contrast, IL-4, IL-6, IL-10, IL-12, TNFα, and CD40L have no or only marginal effects on tetherin expression levels (Blasius et al., 2006; Tavano et al., 2013) and its expression is downmodulated by TGFβ (Sayeed et al., 2013).

Although tetherin expression levels are markedly upregulated upon IFN stimulation, many cell types constitutively express tetherin in the absence of viral infections. Tetherin was originally identified as a marker for bone marrow stromal cells and various tumor cells (Goto et al., 1994; Ishikawa et al., 1995; Ohtomo et al., 1999; Walter-Yohrling et al., 2003; Grützmann et al., 2005; Capurso et al., 2006; Cai et al., 2009). More recently, it has become clear that tetherin is more widely expressed and can be detected to high levels in hepatocytes, pneumocytes, activated T cells, monocytes, pDCs, ducts of major salivary glands, pancreas and kidney cells, vascular endothelium, and many other cell types (Vidal-Laliena et al., 2005; Blasius et al., 2006; Kawai et al., 2006; Erikson et al., 2011). This constitutive expression in many organs and tissues suggests that tetherin is a key player of the early innate immune response. Alternatively, constitutive expression may indicate that tetherin performs cellular functions beyond immunity that require expression also in the absence of IFN and viral infections.

## FUNCTIONS

### INHIBITION OF VIRUS RELEASE

Although tetherin had already been described as an interferon-inducible gene in the 1990s, it took until 2008 to discover the potent antiviral activity of this cellular protein. In this year, two groups reported the ability of tetherin to inhibit the release of budding HIV virions from infected cells (Neil et al., 2008; Van Damme et al., 2008). Electron microscopic analyses revealed that tetherin appears to tether virions to each other as well as to the plasma membrane (**Figure 2B**; Neil et al., 2007, 2008). This antiviral activity depends on the unusual topology of tetherin. Budding virions incorporate one of the two membrane anchors of the restriction factor, whereas the other one remains attached to the plasma membrane of the host cell (Neil et al., 2008; Van Damme et al., 2008; Perez-Caballero et al., 2009). Using sophisticated modified tetherin variants, Venkatesh and Bieniasz (2013) could show that tetherin dimers adopt a parallel configuration with a three- to five-fold preference for the insertion of the GPI-anchor rather than the transmembrane domain into virions. This is in agreement with the observation that HIV progeny virions bud from cholesterol-rich microdomains (Nguyen and Hildreth, 2000; Bhattacharya et al., 2006). Microscopic analyses showed that tetherin accumulates at HIV budding sites with around four to seven molecules per assembly cluster (Habermann et al., 2010; Lehmann et al., 2011). A quantitative Western blotting approach yielded slightly higher numbers suggesting that a few dozen tetherin dimers are used to tether a single virion to the plasma membrane (Venkatesh and Bieniasz, 2013).

Perez-Caballero et al. (2009) showed that an artificial tetherin molecule consisting of the transmembrane domain of the transferrin receptor, the coiled-coil ectodomain of the dystrophia myotonica protein kinase (DMPK), and the GPI modification signal from the urokinase plasminogen activator receptor (uPAR) was able to restrict the release of budding virions. The fact that this artificial protein lacks any sequence homology with tetherin strongly argues against the requirement of any viral or cellular cofactors for viral restriction. Instead, the overall configuration with two membrane anchors and a flexible ectodomain seems to be sufficient to inhibit virion release. As mentioned above, a naturally occuring prion protein form also consists of an N-terminal transmembrane domain, a glycosylated ectodoamin and a C-terminal GPI-anchor. This prion protein variant is expressed at the cell surface and it would certainly be interesting to test whether it displays similar antiviral activities. In agreement with a direct tethering model involving an axial configuration of the dimer, tetherin mutants lacking either the GPI-anchor or the transmembrane domain are non-functional (Neil et al., 2008). Interestingly, several hereditary diseases affect enzymes of the GPI-anchor biosynthetic pathway. The best described probably being paroxysmal nocturnal hemoglobinuria (PNH), a syndrome that is most commonly caused by mutations in phosphatidylinositol glycan A (PIGA). This protein is part of an enzyme complex that catalyzes the first step of the GPI-anchor synthesis, the addition of *N*-acetylglucosamine (GlcNAc) to phosphatidylinositol (PI). Subsequently, GlcNAcPI is deacetylated by the ER-resident enzyme phosphatidylinositol glycan anchor biosynthesis, class L (PIGL). Mutations in PIGL cause the CHIME syndrome that is characterized by colobomas, heart defects, ichthyosiform dermatosis, mental retardation, and ear anomalies (Ng et al., 2012). Although PIGL has been shown to be required for the transport of tetherin to the cell surface (Perez-Caballero et al., 2009), viral infections are not a major symptom of PNH or CHIME syndrome patients.

In agreement with a direct tethering mechanism of the viral membrane to the plasma membrane of the host cell, it has been demonstrated that tetherin is able to inhibit the release of a large number of enveloped viruses. Studies using either virus-like particles or replication competent viruses revealed that tetherin restricts budding of members of alpha-, beta-, gamma-, and deltaretroviruses, lentiviruses, and spumaviruses (Neil et al., 2008; Jouvenet et al., 2009; Groom et al., 2010; Goffinet et al., 2010b), arena- and filoviruses (Jouvenet et al., 2009; Sakuma et al., 2009; Radoshitzky et al., 2010), as well as paramyxo- and rhabdoviruses (Radoshitzky et al., 2010; Weidner et al., 2010; Sarojini et al., 2011; Kong et al., 2012). Interestingly, viruses that bud from intracellular membranes such as HSV-1 or HCoV-229E are also restricted by tetherin (Blondeau et al., 2013; Wang et al., 2014). The effect of tetherin on HCV replication is still controversial (Dafa-Berger et al., 2012; Ye et al., 2012; Pan et al., 2013; Amet et al., 2014). Notably, restriction of virion release is not the only antiviral activity of tetherin. Thus, seemingly discrepant results may be explained by the fact that some of the studies analyzed several rounds of viral replication whereas others focused on the release of viral particles.

### INNATE SENSING AND SIGNALING

Tetherin was identified in an over-expression screening for activators of NF-κB (Matsuda et al., 2003). This raised the possibility that tetherin may also act as signaling molecule in addition to its role as an inhibitor of virion release. Subsequent studies demonstrated that antibody-mediated crosslinking of surface tetherin and – most importantly – virion budding induces the activation of NF-κB (Galão et al., 2012; Tokarev et al., 2013). Thus, tetherin may indeed act as a PRR inducing an antiviral immune response upon binding of budding progeny virions. Notably, however, activation of NF-κB and restriction of virion release are genetically separable functions of tetherin (Tokarev et al., 2013). The presence of the GPI-anchor, for example, is essential for inhibition of virion release but dispensable for signaling. Conversely, disruption of the tetramerization motif specifically disrupts signaling (Tokarev et al., 2013). It remains to be determined whether intrinsic tetherin-mediated activation of NF-κB in the absence of tethered viral particles is just an *in vitro* artifact due to over-expression or plays an important role *in vivo*. In both scenarios (over-expression and sensing of budding virions), tetherin seems to recruit TRAF2 and/or TRAF6 as well as the mitogen-activated kinase TAK1 and TAB, thereby activating the canonical NF-κB pathway (**Figure 2B**; Galão et al., 2012; Tokarev et al., 2013). It is still unclear how exactly these signaling molecules are recruited to tetherin. Although human tetherin contains a putative TRAF binding site [PxExx(Ar/Ac)] in its N-terminal cytoplasmic domain, mutational analyses revealed that this motif is most likely dispensable for TRAF6 recruitment (Ye et al., 2002; Galão et al., 2012). In contrast, mutation of the dual tyrosine motif YxYxxφ abrogated the signaling activity of tetherin. Depletion of AP2 and the analysis of a naturally occurring Y8H variant of tetherin, however, revealed that endocytosis is not required for efficient NF-κB activation (Galão et al., 2012; Sauter et al., 2013). The short isoform of tetherin fails to act as an innate sensor since it lacks the dual tyrosine motif. As this isoform acts in a dominant-negative manner on NF-κB activation, it is probably only homodimers of the long isoform that activate NF-κB (Cocka and Bates, 2012). It is tempting to speculate that the two N-terminal tyrosine residues in homodimers are phosphorylated upon virion sensing to recruit the first components of the NF-κB signaling cascade. Interestingly, a rare single nucleotide polymorphism (SNP) changing arginine at position 19 to histidine also abrogates the signaling activity of tetherin without affecting surface expression or its ability to restrict virion release (Sauter et al., 2013). Unfortunately, this SNP is probably too rare to assess a possible association with disease progression.

Remarkably, tetherin has also been shown to directly interact with the immunoglobulin-like transcript 7 (ILT7, LILRA4, CD85g) on pDCs (Cao et al., 2009; **Figure 2C**). ILT7 forms a complex with FcεRIγ which contains an immunoreceptor tyrosine-based activation motif (ITAM) in its cytoplasmic tail and induces a calcium-dependent signaling cascade that inhibits the release of type I interferons and other proinflammatory cytokines from pDCs (Cao et al., 2006; Cho et al., 2008). Thus, it has been suggested that binding of tetherin to ILT7 may induce a negative feedback signaling to prevent an uncontrolled prolonged inflammatory response (Cao et al., 2009). A recent study by Tavano et al. (2013), however, has challenged this hypothesis. Whereas antibody-mediated crosslinking of ILT7 significantly suppressed IFNα production by pDCs a modulation of IFN production by tetherin was not observed in their experimental setup (Tavano et al., 2013). It is tempting to speculate that binding of ILT7 to tetherin may also activate tetherin-mediated NF-κB signaling.

### STRUCTURAL ORGANIZATION OF THE CELL

In addition to its roles in antiviral immunity tetherin has also been reported to be an organizer of different cellular structures and organelles. It has early been shown that tetherin enters the secretory pathway and is thus mainly transported to the apical membrane of polarized cells (Kupzig et al., 2003). Knockdown experiments revealed that tetherin is required for the maintenance of the apical actin network and microvilli in such cells (Rollason et al., 2007). The protein RICH2 binds to the cytoplasmic dual tyrosine motif of tetherin and to EBP50, thereby linking it to Ezrin and the apical actin cytoskeleton (**Figure 2D**; Rollason et al., 2007). Interestingly, the adaptor protein binding site is masked in this process and RICH2 binding prevents clathrin-mediated endocytosis of tetherin (Rollason et al., 2007). RICH2 is a Rho-type GTPase-activating protein that inhibits the activation of Rac and Rho which is involved in the remodeling of the actin cytoskeleton. The activation of these Rho-GTPases is increased in tetherin-depleted cells (Rollason et al., 2007). Thus, tetherin does not only act as an anchor and stabilizer of the apical actin network but also seems to be involved in the regulation of Rho-GTPases. Interestingly, the *Dictyostelium* protein ponticulin that also contains an N-terminal transmembrane domain and a C-terminal GPI-anchor has also been shown to link the plasma membrane to the cortical actin network (Hitt et al., 1994a,b).

With the GPI-anchor being localized in a lipid raft and the transmembrane domain just adjacent to it, tetherin may also serve as a picket fence stabilizing and organizing membrane microdomains (**Figure 2D**; Kupzig et al., 2003). This hypothesis is supported by the observation of Billcliff et al. (2013) that the N-terminal cytoplasmic tail of tetherin serves as a microdomain-exclusion motif. Thus, tetherin would link membrane rafts to the underlying actin cytoskeleton, a role that has previously been ascribed to the tetraspanin CD82 (**Figure 2D**; Delaguillaumie et al., 2004).

Another interesting observation comes from the hamster ortholog of tetherin called Golgi-resident GPI-anchored protein (GREG). As the name suggests, hamster tetherin is preferentially localized to the Golgi apparatus rather than the plasma membrane. GREG is characterized by a stretch of unique EQ tandem repeats serving as a putative Golgi-retention signal that is absent from all other tetherin orthologs (**Figure 1A**; Li et al., 2007). Nevertheless, the overall topology of the protein is conserved and a certain amount of GREG can also be detected at the plasma membrane (Perez-Caballero et al., 2009). Thus, it is very likely that hamster tetherin is still able to inhibit the release of budding virions despite the presence of a putative Golgi-retention signal. Interestingly, many circular and ring-like structures rather than the classical Golgi cisternae were observed in GREG-depleted cells, suggesting an essential role of GREG in the maintenance of the Golgi complex (**Figure 2E**; Li et al., 2007). A similar phenotype was observed in cells lacking PIGL which is required for the GPI-anchor synthesis (Li et al., 2007). A model has been proposed in which opposing membranes within a cisterna are linked by GREG dimers (**Figure 2E**). These dimers may either stabilize the Golgi structure or act as a sensor, surveilling the distance between opposing membranes. In this context, Swiecki et al. (2011) made the interesting observation that tetherin ectodomain dimers are similar to that formed by BAR-domains. BAR-domains have been shown to bind and stabilize membrane curvatures (Frost et al., 2009). It is tempting to speculate that the extracellular part of tetherin may perform similar activities that are involved in the sensing of budding virions and/or Golgi structure.

## SPECIES-SPECIFIC DIFFERENCES

Tetherin orthologs have been described in many mammalian species (**Figure 1A**). The only non-mammalian tetherin has been identified in the Chinese alligator *Alligator sinensis* (accession numbers: XP_006017475, XP_006017476). The sequence homology to mammalian tetherins is, however, only very limited and it remains to be clarified whether this reptile protein really represents a tetherin ortholog.

Like most antiviral genes, tetherin is under high selection pressure and residues in all three domains have been shown to be under positive selection (Gupta et al., 2009a; McNatt et al., 2009; Lim et al., 2010). Seven very rare non-synonymous SNPs have been described in human tetherin (Y8H, R19H, N49S, D103N, E117A, D129E, and V146L), one of which specifically abrogates the sensing and signaling activity of tetherin (Sauter et al., 2013). A unique characteristic of the human ortholog is the deletion of five amino acids in the cytoplasmic N-terminal tail (**Figure 1A**). Since these five amino acids are also absent from the genomes of the Denisova and Neanderthal, it probably emerged at least 800,000 years ago, before the separation of these ancient hominin species but after the divergence of humans from non-human primates (Sauter et al., 2011b). The methionine residue at position 13 of human tetherin is conserved in many orthologs, suggesting that a long and a short isoform of tetherin are expressed in many mammalian species (**Figure 1A**). Some species, however, such as cats, guinea-pigs, horses, or elephants encode only the short isoform or a variant with deletions in the N-terminal part (**Figure 1A**). One well-characterized example is the cat ortholog, which expresses only the short isoform due to a mutation in the upstream start codon (Celestino et al., 2012). Since this isoform lacks the dual tyrosine motif that is required for activation of NF-κB signaling and binding of RICH2, feline tetherin is probably deficient in these two functions. The short isoform may, however, restrict virion release more efficiently because it is expressed to higher levels at the cell surface due to the lack of the endocytosis signal. Furthermore, a short N-terminal cytoplasmic tail may confer a selective advantage because it reduces the number of target sites for potential cytoplasmic viral antagonists.

Interestingly, most mammalian tetherin orthologs seem not to be able to sense viral particles although they express the long isoform and contain the dual tyrosine motif. Only human tetherin and (to a lesser extent) chimpanzee tetherin have been shown to perform this function (Galão et al., 2012). Although most experiments have been performed in human cells, it has been suggested that the deletion of the five amino acid patch in the cytoplasmic tail of tetherin during human evolution may have led to an increased signaling capacity (Galão et al., 2012). Tetherin orthologs from other species may perform additional functions. As mentioned above, hamster tetherin contains unique EQ tandem repeats that may determine its preferentially intracellular localization and its involvement in the maintenance of the Golgi apparatus (**Figures 1A** and **2E**; Li et al., 2007). The Gray-handed night monkey *Aotus (lemurinus) griseimembra* encodes a tetherin variant that is not able to inhibit virion release. This lack of restriction could be ascribed to a S164T mutation in the extracellular domain of tetherin (**Figure 1A**; Wong et al., 2009). To my knowledge, this is the only naturally occurring tetherin variant that fails to restrict virion release.

Sheep, goats, and cows encode two tetherin variants implying a gene duplication event before the divergence of these ruminants (Arnaud et al., 2010). At least in sheep, both proteins (BST-2A and BST-2B) are able to inhibit the release of budding virions, although they may be differentially expressed in various cell types (Arnaud et al., 2010). Interestingly, BST-2A is characterized by a truncated N-terminal domain and appears to restrict retroviral release more efficiently than BST-2B (**Figure 1A**; Arnaud et al., 2010).

Thus, some tetherin functions such as maintenance of the Golgi structure or innate sensing may have evolved relatively recently during evolution and may hence only be found in few species. In contrast, the inhibition of virion release is conserved among diverse mammals suggesting that this is an ancient function of tetherin.

## VIRAL ANTAGONISTS

### SIV Nef

**FIGURE 3:**
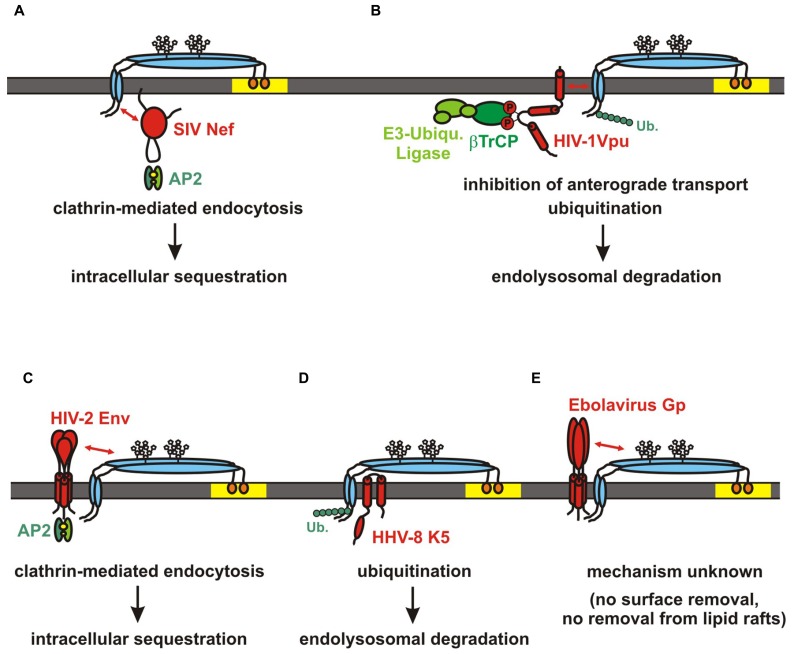
**Viral antagonists of tetherin.** Schematic structure and mode of action are shown for **(A)** SIV Nef, **(B)** HIV-1 Vpu, **(C)** HIV-2 Env, **(D)** HHV-8 K5, **(E)** Ebolavirus Gp. Tetherin is indicated in blue, the viral antagonists are shown in red. SIV Nef and HIV-2 Env sequester tetherin to intracellular compartments without affecting total cellular tetherin levels. In contrast HIV-1 Vpu and HHV-8 K5 induce the ubiquitination and subsequent degradation of the restriction factor. The mechanism of Ebola Gp-mediated tetherin antagonism is still unclear. Interactions between domains of tetherin and its antagonists are indicated by red arrows.

Simian immunodeficiency viruses are primate lentiviruses that have been identified in more than forty different African primate species. Most of these viruses use their accessory protein Nef to counteract tetherin in their respective host species (Jia et al., 2009; Sauter et al., 2009; Zhang et al., 2009; Schmökel et al., 2011). Like many other viral antagonists, Nef enhances virion release by decreasing the surface expression levels of tetherin (**Figure 3A**; Götz et al., 2012; Serra-Moreno et al., 2013). However, total cellular tetherin levels remain unaffected suggesting that Nef sequesters it to intracellular compartments rather than inducing its degradation. Indeed, it has been shown that Nef induces clathrin, AP2- and dynamin2-dependent endocytosis of the restriction factor (**Figure 3A**). Mutational analyses revealed that residues within and adjacent to a highly conserved [D/E]xxxLL motif in the C-loop of Nef are critical for its anti-tetherin activity (Zhang et al., 2011; Serra-Moreno et al., 2011; Götz et al., 2012). Although recruitment of AP2 via this motif is required for Nef-mediated downmodulation of CD4 (Garcia and Miller, 1991; Lindwasser et al., 2008), these mutations specifically disrupted the downmodulation of tetherin (Götz et al., 2012; Serra-Moreno et al., 2013). Thus, residues surrounding the AP2-binding site may be involved in the direct binding of tetherin rather than AP2 recruitment. In agreement with this hypothesis, Serra-Moreno et al. (2013) verified a direct physical interaction between Nef and the N-terminal cytoplasmic tail of tetherin. Interestingly, the sensitivity of tetherin toward Nef maps to the DIWKK motif that is missing in the human ortholog (Jia et al., 2009; Sauter et al., 2009; Zhang et al., 2009). Thus, human tetherin is resistant against Nef-mediated counteraction and may thus represent a hurdle for successful cross-species transmissions of SIV to humans (Sauter et al., 2009, 2010).

### HIV-1 M AND N Vpu

The current AIDS pandemic is a sinister example for the enormous plasticity and adaptability of primate lentiviruses. HIV-1 groups M, N, O, and P are the result of four independent cross-species transmission events from apes to humans (Sharp and Hahn, 2011). SIVcpz and SIVgor, the direct precursors of HIV-1 use their Nef proteins to antagonize the tetherin ortholog of their respective host species. Although human tetherin is resistant to Nef due to a five amino acid deletion in its cytoplasmic tail, pandemic HIV-1 group M strains mastered this species barrier by switching from Nef- to Vpu-mediated counteraction (**Figure 3B**; Sauter et al., 2009). This evolution of Vpu as an effective tetherin antagonist may have been an important prerequisite for the pandemic spread of HIV-1 group M (Sauter et al., 2010). Several residues in the transmembrane domain of Vpu have been shown to directly interact with the transmembrane domain of tetherin (Kobayashi et al., 2011; McNatt et al., 2013). As a consequence, Vpu-mediated counteraction of tetherin is often species-specific (Goffinet et al., 2009; Gupta et al., 2009a; McNatt et al., 2009; Rong et al., 2009). Notably, an AxxxAxxxAxxxW face in the transmembrane domain of HIV-1 group M Vpu is essential for the counteraction of human tetherin (Vigan and Neil, 2010). It remains, however, unclear whether these residues directly interact with the transmembrane domain of tetherin or rather confer stability to the alpha-helical structure of the transmembrane domain (Kleiger et al., 2002; Schneider and Engelman, 2004).

Although an interaction of Vpu with tetherin at the cell surface has been suggested (Iwabu et al., 2009), it is now quite well established that both proteins interact in the TGN (Dubé et al., 2009) and that Vpu inhibits the anterograde transport of tetherin to the plasma membrane (Dubé et al., 2009; Hauser et al., 2010; Andrew et al., 2011; Schmidt et al., 2011). Although this interaction and sequestration to the TGN may be sufficient for a partial relief of restriction, full counteraction activity depends on the presence of a di-serine motif (DSGxxS) in the cytoplasmic part of Vpu that is phosphorylated by casein kinase II (Mangeat et al., 2009; Goffinet et al., 2010a; Schindler et al., 2010). This motif recruits the SCF E3 ubiquitin ligase complex via the adaptor protein βTrCP thereby inducing the subsequent ubiquitination of tetherin. The exact residues in the cytoplasmic tail of tetherin that become ubiquitinated are still unclear (Tokarev et al., 2011; Gustin et al., 2012). The absence of putative ubiquitination sites in the short isoform of tetherin may, however, explain its relative resistance against Vpu (Cocka and Bates, 2012). Although some reports propose an ERAD- and proteasome-dependent degradation of tetherin (Goffinet et al., 2009; Mangeat et al., 2009; Petris et al., 2014), it seems more likely that tetherin enters the ESCRT-dependent endolysosomal pathway upon ubiquitination (Douglas et al., 2009; Iwabu et al., 2009; Mitchell et al., 2009; Janvier et al., 2011; Agromayor et al., 2012; Gustin et al., 2012; Kueck and Neil, 2012; Rollason et al., 2013). Interestingly, the presence of a βTrCP- consensus sequence (DSGxxS) is not absolutely required for efficient anti-tetherin activity (Kluge et al., 2013) and some studies suggest that the di-serine itself rather than βTrCP recruitment is required for Vpu-mediated counteraction of tetherin (Schmidt et al., 2011; Tervo et al., 2011). Whereas Tervo et al. (2011) propose the binding of an as-yet unknown factor to the di-serine it is also conceivable that the presence of this motif is required for the structural integrity of the second alpha-helical domain of Vpu (Coadou et al., 2002, 2003). Notably, this second alpha-helix contains a putative ExxxLV trafficking motif that is required for efficient anti-tetherin activity (Kueck and Neil, 2012) and fusion of this domain to tetherin was sufficient to remove it from the sites of budding (McNatt et al., 2013). A similar DxxxLV motif evolved in the Vpu of a recently isolated highly pathogenic HIV-1 group N strain which counteracted human tetherin as efficiently as pandemic HIV-1 group M Vpus (Sauter et al., 2012). In contrast, most of the previously characterized HIV-1 N Vpus do not contain a DxxxLV motif in their cytoplasmic domain and counteract tetherin only inefficiently (Sauter et al., 2009, 2012). This poor anti-tetherin activity of HIV-1 group N viruses could be a reason for their very limited spread in the human population. Similarly, Vpu proteins of non-pandemic HIV-1 groups O and P have not evolved efficient anti-tetherin activity either (Sauter et al., 2009, 2011a; Yang et al., 2010b; Petit et al., 2011). Thus, only pandemic HIV-1 M strains mastered the tetherin hurdle “perfectly” by switching from Nef to Vpu to antagonize the human ortholog of this restriction factor.

### HIV-2 AND SIV Env

The second human immunodeficiency virus HIV-2 is the result of at least nine independent cross-species transmissions of SIVsmm infecting sooty mangabeys to humans (Sharp and Hahn, 2011; Ayouba et al., 2013). These transmission events gave rise to HIV-2 groups A–I. HIV-2 does not encode a *vpu* gene and switched from Nef to its envelope protein (Env) to antagonize human tetherin (Le Tortorec and Neil, 2009). Similar to Nef-mediated counteraction of tetherin, Env does not induce the degradation of this restriction factor but rather sequesters it to intracellular compartments, probably the TGN (**Figure 3C**; Le Tortorec and Neil, 2009; Hauser et al., 2010). The interaction with tetherin occurs very likely via the ectodomain of Env (Lopez et al., 2010) and depends on an endocytic motif in gp41 (Le Tortorec and Neil, 2009). Notably, most assays were performed with Env alleles from HIV-2 group A strains and it remains unclear whether other HIV-2 groups also evolved Env-mediated anti-tetherin activity and/or whether the ability to antagonize tetherin correlates with the spread of the respective HIV-2 group in the human population.

### OTHERS

The broad antiviral activity of tetherin is reflected by the fact that a substantial number of enveloped viruses have evolved antagonists of this restriction factor. Similar to the retroviral proteins mentioned above, most antagonists reduce tetherin levels at the sites of budding to enable efficient release of progeny virions.

The Herpes simplex virus 1 (HSV-1) glycoprotein M (gM), the Chikungunya virus non-structural protein 1 (Nsp1) and the Env of SIVtan and equine infectious anemia virus (EIAV), for instance, all reduce the surface expression levels of tetherin (Gupta et al., 2009b; Blondeau et al., 2013; Jones et al., 2013; Yin et al., 2014). Neuraminidase (N1 and N2) has been suggested to be the tetherin antagonist of influenza viruses (Yondola et al., 2011; Mangeat et al., 2012; Leyva-Grado et al., 2013) and Sendaivirus uses the fusion (F) and hemagglutinin-neuraminidase proteins (HN) in concert to induce the degradation of the restriction factor (Bampi et al., 2013).

In 2006, tetherin was identified in a screen for factors that are downmodulated by the RING-CH ubiquitin ligase K5 of the Kaposi’s sarcoma-associated herpesvirus (KSHV, HHV-8; Bartee et al., 2006). It soon became clear that HHV-8 utilizes K5 to ensure efficient virion release by inducing the degradation of tetherin. K5 ubiquitinates lysine 18 in the cytoplasmic tail of tetherin thereby targeting it for proteasomal or ESCRT-dependent endo-lysosomal degradation (**Figure 3D**; Mansouri et al., 2009; Pardieu et al., 2010; Agromayor et al., 2012). In agreement with an ongoing coevolution between tetherin and its viral antagonists, K5 efficiently counteracts human tetherin but fails to antagonize the rhesus macaque and mouse orthologs (Pardieu et al., 2010). Bartee et al. (2006) made the interesting observation that MARCH-VIII, the cellular homolog of K5 is also able to induce the degradation of tetherin.

In contrast to other tetherin antagonists, the Ebolavirus glycoprotein (Gp) is able to enhance virion release without decreasing the surface levels of the restriction factor (**Figure 3E**; Lopez et al., 2010; Kühl et al., 2011). Interestingly, removal of tetherin from lipid rafts is not involved either (Lopez et al., 2010) and Ebolavirus Gp fails to rescue the release of arenaviruses (Radoshitzky et al., 2010). Although the exact mechanism of Gp-mediated counteraction of tetherin remains unclear, it has been suggested that tetherin interacts directly with the GP2 subunit (Kühl et al., 2011). A similar mechanism has also been proposed for FIV Env. Morrison et al. (2014) showed that FIV Env incorporation is required to antagonize feline tetherin but does not involve a reduction of total or surface tetherin levels. Like Ebola Gp, FIV Env was not able to rescue the release of non-cognate particles (Celestino et al., 2012; Morrison et al., 2014). However, another study suggested that FIV does not encode a direct antagonist but rather overcomes restriction by direct cell-to-cell spread (Dietrich et al., 2011).

Thus, viruses may also evolve evasion strategies without directly targeting tetherin. It has for example been suggested that some viruses do not induce IFN production *in vivo* or have evolved means to inhibit the IFN-mediated expression of tetherin (Lim and Emerman, 2009; Mangeat et al., 2012). Influenza virus for instance does not only use its Neuraminidase protein to directly antagonize tetherin but may also impede the induction of tetherin via the viral protein NS1 (Mangeat et al., 2012). To evade restriction some viruses such as HCV may also bud from internal membranes that contain no or only low levels of tetherin. Similarly, direct cell-to-cell spread has been suggested to be used by some viruses to overcome restriction (Jolly et al., 2010; Dietrich et al., 2011; Ilinskaya et al., 2013). Furthermore, the antiviral activity of tetherin is simply saturated if a large number of virions is budding (Yadav et al., 2012). Some viruses may even exploit tetherin for their own benefit: HTLV-1 infected cells produce tetherin-containing extracellular viral assemblies that are transferred to neighboring cells and are required for efficient spread of infection (Pais-Correia et al., 2010). Tetherin has also been suggested to enhance the entry of human cytomegaloviruses and to be required for efficient FIV particle release (Viswanathan et al., 2011; Morrison et al., 2014). In summary, viruses have evolved a multiplicity of mechanisms to counteract, evade or even hijack the restriction imposed by tetherin.

## SUMMARY AND CONCLUDING REMARKS

The continuous arms race between viruses and their hosts has certainly driven the evolution of the host restriction factor tetherin. Whereas the ability of tetherin to restrict virion release seems to be an ancient function that is highly conserved among all mammalian orthologs, some species have evolved unique features of this protein. Bovids, for instance, express two tetherin homologs that probably differ in their expression pattern and may thus facilitate adaptation to emerging viral infections (Arnaud et al., 2010). Human and (to a lesser extent) chimpanzee tetherin are apparently the only tetherin orthologs that act as innate sensors activating the NF-κB signaling cascade upon binding of viral particles (Galão et al., 2012). Conversely, tetherin also exerted a substantial selection pressure on viral evolution. Diverse enveloped viruses have evolved effective ways of escaping restriction. Whereas some viral proteins directly target tetherin and sequester it away from the sites of budding and/or induce its degradation, other viruses may overcome restriction via cell-to-cell spread or prevent the mounting of an IFN response to inhibit expression of tetherin and other ISGs.

The AIDS pandemic is an impressive example of this ongoing coevolution between viruses and tetherin. Analyses of different HIV-1 groups revealed that counteraction of tetherin may be a prerequisite for the efficient spread of lentiviruses in the human population (Sauter et al., 2010): in contrast to pandemic HIV-1 group M viruses, non-pandemic HIV-1 groups N, O, and P strains failed to evolve efficient tetherin antagonists after cross-species transmissions of SIV to humans (Sauter et al., 2009, 2011a). Notably, however, patients infected with non-pandemic HIV-1 strains develop high viral loads and ultimately progress to AIDS although tetherin is not efficiently counteracted (Ayouba et al., 2000; Plantier et al., 2009; Vessière et al., 2010; Delaugerre et al., 2011). This strongly suggested that the evolution of a specific tetherin antagonist is required for efficient viral transmission and spread of the virus in the population rather than for efficient replication within an infected individual. In agreement with this, the presence of Vpu did not or only slightly enhance the cytopathicity of HIV-1 in humanized mouse models (Aldrovandi and Zack, 1996; Sato et al., 2012). Furthermore, Vpu boosted the initial phase of R5- and X4-tropic HIV-1 replication but was less important for viral dissemination during late stages of infection (Aldrovandi and Zack, 1996; Sato et al., 2012; Dave et al., 2013). These observations would be in agreement with a model in which cell-free virions are involved in transmission of the virus, whereas spread within patients occurs mainly via direct cell-to-cell spread that overcomes tetherin restriction (Jolly et al., 2010; Sato et al., 2012).

A recent publication by Pickering et al. (2014), however, challenged this hypothesis revealing that potent anti-tetherin activity is not only a characteristic of transmitted/founder viruses but preserved throughout infection. Thus, efficient counteraction of tetherin may confer a selective advantage during all stages of viral infection. One possible explanation for this observation is that tetherin may still restrict direct cell-to-cell spread of HIV-1. In contrast to Jolly et al. (2010), Kuhl et al. (2010) reported that tetherin reduces the transfer of infectious material via virological synapses. Similarly, Casartelli et al. (2010) also showed that tetherin is able to restrict direct cell-to-cell spread. They suggest that tetherin induces the transfer of large patches of cross-linked viruses with reduced infectivity from producer to target cells (Casartelli et al., 2010). Alternatively, the contribution of cell-free virions to persistant HIV-1 infection may be higher than initially thought. This hypothesis is supported by the successful use of broadly neutralizing antibodies in recent vaccine trials (Klein et al., 2013). Furthermore, different functions of tetherin may exert selection pressure at different stages of the viral replication cycle. Thus, even if restriction of virion release primarily affects transmission efficiency and early viral dissemination, the sensing activity of tetherin (Galão et al., 2012) may still exert a high selection pressure on Vpu function during late stages of infection. In addition to its restriction and signaling functions, tetherin seems to enhance the antibody opsonization of infected cells by increasing the accessibility of epitopes on the cell surface (Alvarez et al., 2014; Pham et al., 2014). As a result, Vpu-mediated down-modulation of tetherin may confer a selective advantage throughout all stages of infection because it reduces the susceptibility of infected cells to NK-cell-mediated antibody-dependent cellular cytotoxicity (Alvarez et al., 2014; Pham et al., 2014).

Several additional mouse studies affirmed a crucial role of tetherin in restricting viral infection *in vivo*. In contrast to the humanized mouse models described above, these studies focused on the infection of immunocompetent mice with murine pathogens. In a study by Liberatore and Bieniasz (2011), viral loads were significantly increased in tetherin knockout mice infected with Moloney MLV and these mice progressed to disease faster than their wild type littermates. Increased replication of Moloney MLV in tetherin-deficient mice was also confirmed by a study of Swiecki et al. (2012). Paradoxically, however, viral titers in the lungs were reduced upon infection with vesicular stomatitis virus in this mouse model (Swiecki et al., 2012). A third *in vivo* study took advantage of a polymorphism that disrupts the first start codon of tetherin (Barrett et al., 2012). As a consequence, these mice express only the short form of tetherin which lacks the tyrosine-based endocytosis signal and is therefore expressed to higher levels at the cell surface. In agreement with the other two studies, the increased tetherin expression resulted in decreased viral loads and reduced the pathogenic effects of Friend MLV (Barrett et al., 2012).

The observation that tetherin knockout mice do not show any obvious developmental or functional defects (Liberatore and Bieniasz, 2011) argues against essential cellular roles of tetherin beyond antiviral immunity. Some activities of tetherin such as the ability to induce the NF-κB signaling cascade may, however, be unique for the human ortholog. Thus, mouse models cannot fully recapitulate all characteristics of human tetherin and further studies are warranted to characterize the relative contribution of different tetherin functions in antiviral activity and beyond.

## Conflict of Interest Statement

The author declares that the research was conducted in the absence of any commercial or financial relationships that could be construed as a potential conflict of interest.
